# Editorial: Studying the immune microenvironment of liver cancer using artificial intelligence

**DOI:** 10.3389/fimmu.2025.1679921

**Published:** 2025-08-26

**Authors:** Da-ke Zhang, Jie-liang Chen, Bo-ran Yang, Bing Yang

**Affiliations:** ^1^ Key Laboratory of Biomechanics and Mechanobiology, Ministry of Education, Beijing Advanced Innovation Center for Biomedical Engineering, School of Engineering Medicine, Beihang University, Beijing, China; ^2^ Shanghai Medical College, Fudan University, Shanghai, China; ^3^ Tianjin Wutong High School, Tianjin, China; ^4^ Department of Cell Biology, College of Basic Medical Sciences, Tianjin Medical University, Tianjin, China; ^5^ Department of Public Health, International School, Krirk University, Bangkok, Thailand

**Keywords:** artificial intelligence - AI, liver disease, immunotherapy, hepatocellular carcinoma, cancer immune microenvironment, cancer precision medicine

A significant number of deaths each year can be attributed to liver cancer, which is known for its rapid progression and poor prognosis (1). It has a severe impact on quality of life and continues to present a major global public health challenge.

**Figure 1:**
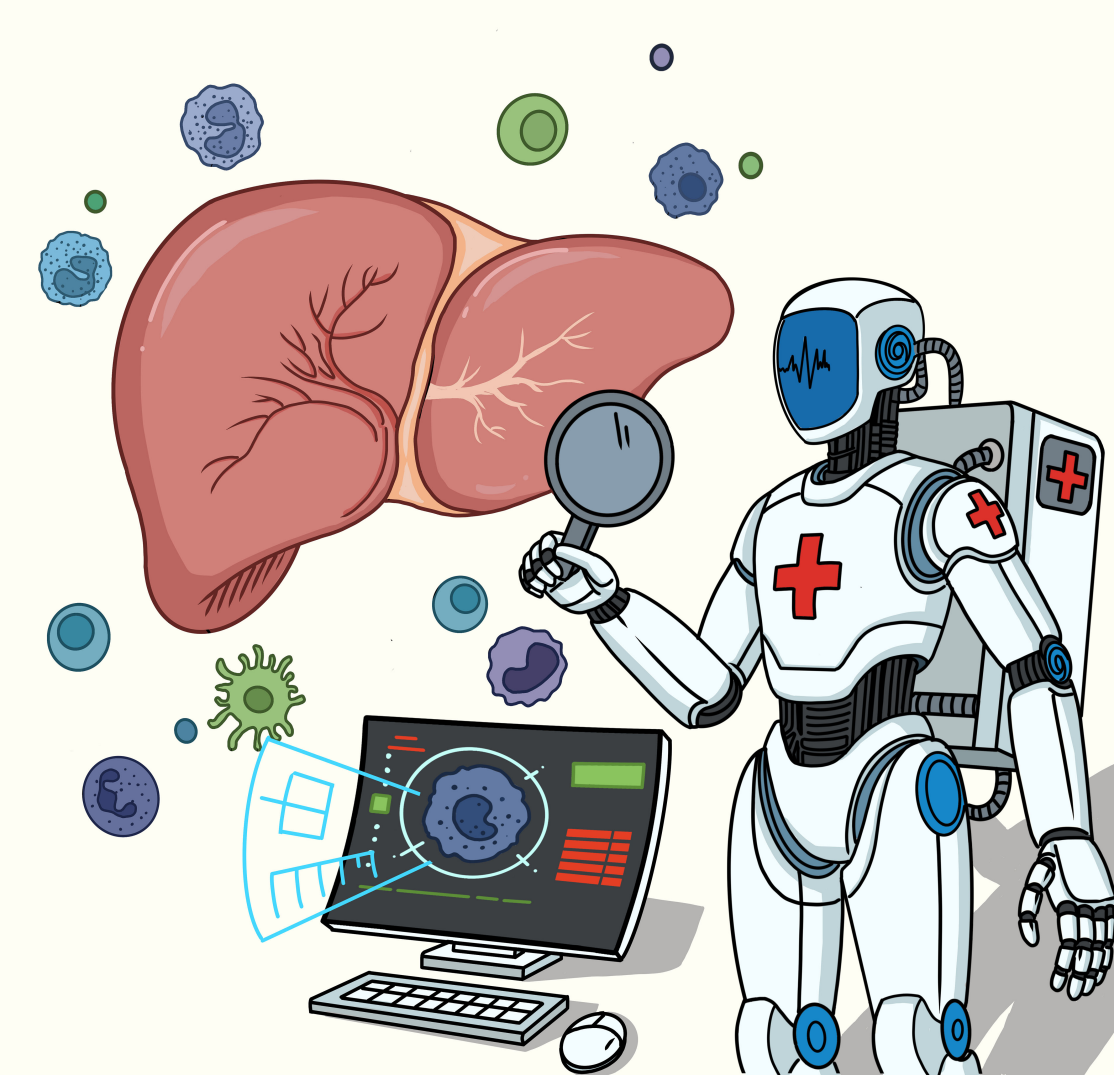
Amazing helping hand: Artificial Intelligence in immunological management of liver disease.

Liver cancer often renders chemotherapy and radiation ineffective, complicating treatment. The need for more effective treatment options has led to pioneering technologies being investigated, including artificial intelligence (**Figure 1**).

Cancer precision medicine aims to ensure treatments work as well as possible for each patient, while also trying to reduce any nasty side effects. Significant progress in oncology has been made by artificial intelligence through high-dimensional datasets and computing/deep learning (2, 3).

Nevertheless, the use of artificial intelligence in medicine is still in its infancy (4). Despite rapid advancements in algorithmic development, significant challenges remain in the areas of clinical data accumulation, data standardization, and quality verification. The accuracy of artificial intelligence in clinical practice still needs to be improved.

The present editorial introduces the compendium of articles that have been published in Frontiers in Immunology: Cancer Immunity and Immunotherapy Research Topic. We hope that this will encourage high-quality research on artificial intelligence in the field of relativity.

*Harnessing multi-omics and artificial intelligence: revolutionizing prognosis and treatment in hepatocellular carcinoma* by Wang et al. The study gained an understanding of the different types of this cancer, improving prediction and treatment by combining different kinds of data.

*Machine learning-driven prediction of immune checkpoint inhibitor responses against cholangiocarcinoma: a bile biopsy perspective* by Zhang et al. Research aims to develop models to detect and treat cholangiocarcinoma early.

*Preoperative assessment of liver regeneration using T1 mapping and the functional liver imaging score derived from Gd-EOB-DTPA-enhanced magnetic resonance for patient with hepatocellular carcinoma after hepatectomy* by Li et al. The author proves that T1 mapping parameters and functional liver imaging score are potential non-invasive indicators of liver regeneration.

*Multiomic analysis of lactylation and mitochondria-related genes in hepatocellular carcinoma identified MRPL3 as a new prognostic biomarker* by Xing et al. The author demonstrates that MRPL3 is a dependable predictive biomarker in diagnosing and treating hepatocellular carcinoma.

*Pinpointing the integration of artificial intelligence in liver cancer immune microenvironment* by Bukhari et al. This review covers recent progress in the immune microenvironment of hepatocellular carcinoma using artificial intelligence.

*Integrative multi-omics analysis reveals a novel subtype of hepatocellular carcinoma with biological and clinical relevance* by Li et al. This study has built an effective model to predict outcomes for patients with this type of cancer and identified new subgroups.

*The complex role of immune cells in antigen presentation and regulation of T-cell responses in hepatocellular carcinoma: progress, challenges, and future directions* by Ning et al. This review gives the latest information about this field by studying how liver cancer antigen presentation works.

*Screening of genes co-associated with osteoporosis and chronic HBV infection based on bioinformatics analysis and machine learning* by Yang et al. The study also focuses on diagnosing and treating chronic HBV. New insights have been gained into the relationship between osteoporosis and chronic HBV infection.

*Lactylation signature identifies liver fibrosis phenotypes and traces fibrotic progression to hepatocellular carcinoma* by Li et al. This research focuses on hepatocellular carcinoma arising from liver fibrosis, particularly lactylation and related immune infiltration.

*Causal relationship between immune cell phenotypes and risk of biliary tract cancer: evidence from Mendelian randomization analysis* by Hu et al. Mendelian randomization was employed in this study to explore the potential association between immune cell phenotypes and biliary tract cancer.

*Hepatitis B-related hepatocellular carcinoma: classification and prognostic model based on programmed cell death genes* by Tian et al. This study used various bioinformatics techniques to analyze RNA sequencing data from patients with Hepatitis B - hepatocellular carcinoma. A prognostic model was also developed, based on genomic and clinical information.
